# Microstructural changes of white matter assessed with diffusional kurtosis imaging in extremely preterm infants with severe intraventricular hemorrhage

**DOI:** 10.3389/fped.2022.1054443

**Published:** 2022-12-20

**Authors:** Li-Min Guo, Meng Zhao, Yue Cai, Na Li, Xiao-quan Xu, Xuan zhang, Jiu-Lou Zhang, Qi-Lian Xie, Si-si Li, Xiao-Qing Chen, Shu-Dong Cui, Chao Lu

**Affiliations:** ^1^ Department of Pediatrics, The First Affiliated Hospital of Nanjing Medical University, Nanjing, China; ^2^ Department of Radiology, The First Affiliated Hospital of Nanjing Medical University, Nanjing, China; ^3^ Department of Critical Care Medicine, Anhui Children's Hospital, Hefei, China; ^4^ Clinical Laboratory, Children's Hospital of Zhejiang University School of Medicine, Hangzhou, China

**Keywords:** intraventricular hemorrhage, extremely preterm infants, white matter, diffusional kurtosis imaging, mean kurtosis, microstructural changes

## Abstract

**Objective:**

Intraventricular hemorrhage (IVH) is a serious neurological complication in premature infants. This study aimed to investigate the white matter impairments and neurodevelopmental outcomes of severe IVH in extremely preterm infants with gestation age less than 28 weeks.

**Methods:**

We retrospectively evaluated the extremely preterm infants between 2017 and 2020. Neurodevelopmental outcomes were evaluated with the Bayley Scales of Infant and Toddler Development-III at 2 years of corrected age. Diffusional kurtosis imaging (DKI) was employed to evaluate the microstructural changes in white matter tracts. Mean kurtosis (MK) and fractional anisotropy (FA) values of DKI were measured in the brain regions including posterior limbs of the internal capsule (PLIC) and the corpus callosum at term equivalent age.

**Results:**

Of 32 extremely preterm infants with severe IVH during the follow-up period, 18 cases were identified as neurodevelopmental impairments. The delay rates of motor and language were 58.4% and 52.7%. The cases with neurodevelopmental impairments had lower MK and FA values in both bilateral PLIC and the corpus callosum. The analysis of multivariable regression models predicting motor and language outcomes at 2 years of corrected age, showed that the decreases of MK values in both PLIC and the corpus callosum at the term equivalent age contributed to a significantly increased risk of neurodevelopmental impairments (all *p *< 0.05). During follow-up period, obvious loss of nerve fiber bundles was observed with DKI tractography.

**Conclusion:**

Motor and language abilities at age 2 years were associated with MK values of DKI at the term equivalent age in both PLIC and the corpus callosum of extremely preterm infants with severe IVH. The evaluation of white matter microstructural changes with MK values might provide feasible indicators of neurodevelopmental outcomes of extremely preterm infants with severe intraventricular hemorrhage.

## Introduction

The gestational ages of extremely preterm infants (EPI) are less than 28 weeks. Extremely preterm infants are extremely susceptible to all kinds of brain injury, such as intraventricular hemorrhage (IVH), post hemorrhagic hydrocephalus and white matter injury. As a serious neurological complication, intraventricular hemorrhage might result in acute stage death and future neurodevelopmental disabilities (1–3).

It was well documented that neurodevelopmental impairments were found in premature infants comprising delays of motor and language. White matter impairment resulted from brain hemorrhage is an important cause (4, 5). Periventricular regions are particularly sensitive to hemorrhage and post-hemorrhagically oxidative stress-related injuries (6), which lead to white matter failing to mature and myelinate properly in the premature brains. Disorder of posterior limb of the internal capsule (PLIC) is related to a serial of neurological disabilities, including the impairment of gross motor ability in preterm children at the age of four years (7). The corpus callosum is an important white matter structure that connects the left and right brain hemispheres. Early in life, brain injury may cause maldevelopment and growth retardation of corpus callosum which may lead to severe motor delay and cerebral palsy at the age of two years (7). Many studies also have validated the association between language ability at age 2 years and the function of corpus callosum in premature infants (4, 8). However, a favorable and effective assessment method for impairments of brain white matter in extremely premature infants suffered to severe intraventricular hemorrhage is still deficient.

Microstructural changes of brain white matter, such as fiber bundle integrity and myelination may be evaluated with a magnetic resonance imaging (MRI) technique, diffusion tensor imaging (DTI). Previous studies from DTI metrics demonstrated abnormal changes of white matter microstructure were associated to motor impairment in very preterm neonates with high-grade brain injury (9). But DTI technology is defective as well. It is based on the assumption that water molecules diffuse freely and that diffusion only reflects the Gaussian distribution. It is unable to completely characterize tissue microstructure (10, 11).

Diffusion kurtosis imaging (DKI) is an extensional technique of DTI. DKI reflects the non-Gaussian water diffusion. As a novel diffusion-weighted technique of MRI, DKI may provide sensitive and comprehensive measures for the quantitative evaluation of microstructural changes of brain white matter (10–12). DKI exhibited improved specificity in detecting pathological changes in the developing brain (10, 11). DKI may provide both diffusion metrics, such as fractional anisotropy (FA), as well as kurtosis parameters, including mean kurtosis (MK), which is specific parameter that cannot be expressed by DTI. FA reflects the degree of anisotropy of water molecules in nerve tissues and can indicate decreased white matter integrity caused by hypomyelination. MK value was more sensitive in detecting the microstructural change of white matter. Decreased MK could reflect the demyelination of brain white matter, the loss of neuron and increased extracellular space resulted from brain hemorrhagic injury (10–12).

White matter tract integrity can characterize brain microstructure in areas with highly aligned fiber bundles. DTI-based fiber tractography is routinely used in clinical applications to visualize major white matter tracts. However, DTI is limited due to its capability of resolving intra-voxel multi-fiber populations. Sophisticated models often require long acquisition times. DKI combines sophisticated modeling of the diffusion process with short acquisition times. For the corticospinal tract, significantly larger tract volumes were seen in DKI-based fiber tractography. DKI-based fiber tractography contributes to advanced visualization under clinical time constraints (13, 14). Therefore, DKI is sensitive to different microstructural properties and is a useful complementary technique to more fully investigate white matter (15).

The purpose of this study was to evaluate microstructural changes of white matter by DKI technology and to investigate related neurodevelopmental impairments in extremely preterm infants with severe IVH.

## Materials and methods

### Patients

The study population comprised all extremely preterm infants admitted to the Neonatal Intensive Care Units between January 1, 2017 and October 1, 2020. The study design was approved by the local ethics committee. Inclusion criteria were: preterm infants with gestational age <28 weeks, informed parental consent. Infants with congenital abnormality were excluded.

IVH on ultrasound scan was graded by utilization of the Papile classification: Grade I, hemorrhage limited to the subependymal germinal matrix. Grade II, Blood within ventricular system without the lateral ventricular dilatation. Grade III, hemorrhage with the lateral ventricular dilatation. Grade IV, Blood within the ventricular system and parenchyma (16). Mild IVH group includes Grade I and II. Severe IVH was defined as Grades III or IV. All of neonates with IVH were diagnosed with cranial ultrasound within the first 3 days of life and subsequent 1 week. The patients were followed-up every three months for the first one year after discharge from the hospital, then every six months for the subsequent two years. Neurodevelopmental outcomes were evaluated with the Bayley Scales of Infant and Toddler Development-III (Bayley-III) at 2 years of corrected age. Cases were divided into neurodevelopmental impairments group and non-neurodevelopmental impairments group.

MRI was performed using a Siemens MR scanner. DKI scans were performed at 37–42 weeks postmenstrual age (PMA) and during the subsequent followed-up period. The diffusion directions = 20, and the b-values = 0, 1000, and 2000 s/mm^2^. The TR/TE was 6800/118 ms for both b = 1000 s/mm^2^ and b = 2000 s/mm^2^ in this DKI sequence. The acquisition matrix was 128 × 130. The reconstruction matrix was 256 × 256. The slice thickness was 3 mm. The field of view (FOV) was 240 mm × 240 mm × 240 mm. The total DKI scan time was 538 s. All raw DKI data were processed using DIPY imaging library in Python (https://dipy.org/). The parametric maps of MK and FA were then generated. Regions of interest (ROIs) were manually delineated on transverse slices using ITK-SNAP software (http://www.itksnap.org/pmwiki/pmwiki.php), and then automatically projected onto the other two parametric maps. Some different anatomical WM structures were investigated, including the left and right posterior limbs of the internal capsule, the genu and splenium of the corpus callosum. Measurements were obtained by an experienced neuroradiologist who was blinded to the clinical information. The ROI size for each subject was identical for the left and right hemispheres. ROIs were placed at the center of structure of WM to minimize variation (Supplementary Figure S1). To evaluate intra-observer reliability, the neuroradiologist performed the same measurements for randomly selected preterm infants 8 weeks later to avoid recall bias. To evaluate inter-observer reliability, another neuroradiologist who was also blinded to the clinical information performed the same measurements for the same neonates (10).

Tractography was performed with a fiber tracking program, Neuro 3D (MR) in Siemens MR system. The tracts through the left and right PLIC and the CC were identified primarily by the color-coded FA map. For both PLIC bundles, one ROI was placed on an axial slice at the level of the foramen of Monro and the second on the adjacent slice above this landmark. For the CC bundle, two ROIs were placed on sagittal slices around the midplane of the CC. All fibers passing through both ROIs were traced. For these traced bundles, different fiber tracking parameters were extracted, including FA, MK, and volume and length of the fiber bundles. The volume of the bundle was defined as the volume of all voxels through which one or more fibers passed, and the average length of the bundle was defined as the average length of all fibers included in the bundle.

To avoid image distortion in diffusion images resulted from IVH, especially in those with severe IVH lesions, Readout segmentation of long variable echo-trains (RESOLVE) technology was used to increased image quality and higher lesion conspicuity as described previously (17).

Similar to that used in previous studies (4, 18, 19), we used a composite variable, MedRisk, to analyze the contributions of number of medical complications to neurodevelopmental impairments. These medical complications included necrotizing enterocolitis, pulmonary hemorrhage, sepsis, patent ductus arteriosus and retinopathy. The levels of risk were divided into low risk (0 risk factors), medium risk (1 or 2 risk factors), and high risk (≥3 risk factors).

### Statistical analyses

Statistical analysis was performed using SPSS software. Independent sample t-test and Pearson *x*^2^ test was used for comparison of quantitative and qualitative variables between the two study groups. A *p*-value of 5% or lower was considered statistically significant. Receiver operating characteristic curves (ROC) analysis was performed. The area under the ROC curve (AUC) and cut-off values were calculated. As described previously (4), some hierarchical linear regression models were conducted to evaluate relationships between neurodevelopmental scores from Bayley-III scale and MK values of DKI in both the PLIC and corpus callosum. Postmenstrual age (PMA) and MedRisk scores were entered in the models in the first step to ensure results were related to white matter microstructure metrics rather than patients age at scan or medical risk. Next, the main statistical effects of the white matter metrics including MK values in both the PLIC and corpus callosum were assessed. To determine if the number of medical complications moderated the prediction of neurodevelopmental outcomes based on MK, we then entered MedRisk as an interaction term within the model.

To obtain further evidence, the variables found to be significantly (*p* < 0.05) associated with the dependent variable in the univariate analysis were entered in a stepwise manner into a multivariate logistic regression analysis. The simultaneous effect of different variables and their calculated Odds ratios (OR) and 95% confidence intervals (CI) were then assessed.

## Results

In total, 7,224 infants were admitted to the NICU between January 1, 2017 and October 1, 2020. Of them, 98 extremely preterm infants with the gestational age less than 28 weeks fulfilled the inclusion criteria in this study. The overall frequency of grade I, II, III and IV IVH was respectively 5.1% (5 cases), 62.2% (61 cases), 19.4% (19 cases), and 13.3% (13 cases). All of 32 cases with severe intraventricular hemorrhage (grades III and IV) were followed for more than 2 years. Among the severe cases, 18 cases were identified as neurodevelopmental impairments with Bayley-III Scale at 2 years of corrected age. The rates of motor and language delay were 58.4% and 52.7%. The 32 extremely preterm infants were divided into two subgroups, neurodevelopmental impairments group and non-neurodevelopmental impairments group. The motor scores were 63.49 ± 9.12 and 80.19 ± 13.55 (*p* < 0.01) respectively. The language scores were also lower in neurodevelopmental impairments group (67.43 ± 9.46 vs. 81.49 ± 17.21; *p* < 0.01).

The DKI-derived parameters showed good inter-observer and excellent intra-observer reliabilities for all selected ROIs (using the right and left PLIC, the genu and splenium of the corpus callosum as examples in Supplementary Table S1). Compared with non-neurodevelopmental impairments group, the cases with neurodevelopmental impairments at 2 years of corrected age had lower MK and FA values in both bilateral PLIC and the corpus callosum at term equivalent age (37–42 weeks of postmenstrual age) (Table 1). ROC analysis revealed that the cut-off values of MK were 0.38, 0.36, 0.44 and 0.42 in the left and right PLIC, the genu and splenium of corpus callosum respectively for the prediction of neurodevelopmental impairments and the AUC was 0.802, 0.827, 0.766 and 0.764 (all *p* < 0.05, Supplementary Figure S2).

**Table 1:** Comparison of DKI parameters in PLIC and corpus callosum between two groups of neurodevelopmental impairments and non-neurodevelopmental impairments.

			Neurodevelopmental impairments (*n* = 18)	Non-neurodevelopmental impairment (*n* = 14)	*t*	*p* value
PLIC	Left	FA	0.28 ± 0.05	0.57 ± 0.05	15.99	<0.001
		MK	0.36 ± 0.06	0.57 ± 0.03	12.67	<0.001
	Right	FA	0.28 ± 0.04	0.42 ± 0.07	6.85	<0.001
		MK	0.34 ± 0.09	0.59 ± 0.03	10.01	<0.001
CC	Genu	FA	0.28 ± 0.05	0.58 ± 0.09	11.95	<0.001
		MK	0.42 ± 0.11	0.49 ± 0.07	2.28	0.030
	Splenium	FA	0.38 ± 0.02	0.58 ± 0.11	8.32	<0.001
		MK	0.41 ± 0.17	0.77 ± 0.08	7.63	<0.001

Data are represented as mean ± Standard Deviation or Number of cases (%). IVH, intraventricular hemorrhage; PLIC, posterior limb of the internal capsule; CC, corpus callosum; FA, fractional anisotropy; MK, mean kurtosis.

Table 2 provides the results of multivariable linear regression models predicting motor outcomes at 2 years of corrected age from MK values in both the bilateral PLIC and the corpus callosum, beyond variation accounted for by PMA. MK values of the left PLIC contributed to motor impairment (Model 2), explaining an additional 24% (*p *< 0.01) of the variance above that accounted for by PMA and MedRisk alone (Model 1). 34% of the variance in motor was accounted for by the model. The interaction term did not contribute additional variance (Model 3), indicating that the association between motor outcomes at 2 years of corrected age and MK of the left PLIC in infancy was similar for children regardless of the number of MedRisk conditions. Consistently, the decreases of MA values of the right PLIC, the genu and splenium of the corpus callosum also contributed to motor impairments (Model 5, 8 and 11), explaining an additional 19%, 16% and 15% of the variance above that accounted for by PMA and MedRisk alone respectively (Model 4, 7 and 10, all *p *< 0.05). While the interaction terms did not contribute additional variances in Model 6, 9 and 12.

**Table 2:** Prediction of bayley-III motor scores at 2 years of corrected-age from tract MK values of PLIC and corpus callosum beyond covariates of post menstrual age at DKI scan (PMA scan) and neonatal medical complications (medRisk).

		Model 1	Model 2	Model 3
PMA Scan	B (SE)	1.33 (1.17)	2.35 (1.66)	1.85 (1.79)
MedRisk	B (SE)	−6.31 (4.16)	−5.68 (3.63)	−11.69 (7.89)
PLIC-L	B (SE)	–	44.09 (13.75)	25.30 (11.11)
PLIC-L × MedRisk	B (SE)	–	–	−14.47 (9.32)
	R^2^-change	–	0.24**	0.01
	Total R^2^	0.10	0.34**	0.35**
	Adjusted R^2^	0.04	0.27	0.26
		Model 4	Model 5	Model 6
PMA Scan	B (SE)	1.33 (1.17)	2.28 (1.72)	2.63 (1.78)
MedRisk	B (SE)	−6.31 (4.16)	−2.61 (1.91)	6.87 (3.03)
PLIC-R	B (SE)	–	45.78 (16.53)	74.97 (29.66)
PLIC-R × MedRisk	B (SE)	–	–	−21.41 (10.04)
	R^2^-change	–	0.19*	0.02
	Total R^2^	0.10	0.29*	0.31*
	Adjusted R^2^	0.04	0.22	0.21
		Model 7	Model 8	Model 9
PMA Scan	B (SE)	1.33 (1.17)	2.56 (1.79)	2.58 (1.95)
MedRisk	B (SE)	−6.31 (4.16)	−6.61 (3.83)	−6.29 (3.71)
CC-G	B (SE)	–	41.84 (16.74)	42.99 (10.72)
CC-G × MedRisk	B (SE)	–	–	−0.79 (0.33)
	R^2^-change	–	0.16*	0.01
	Total R^2^	0.10	0.26*	0.27*
	Adjusted R^2^	0.04	0.19	0.17
		Model 10	Model 11	Model 12
PMA Scan	B (SE)	1.33 (1.17)	1.73 (0.83)	1.68 (0.85)
MedRisk	B (SE)	−6.31 (4.16)	−1.99 (0.95)	22.39 (12.21)
CC-S	B (SE)	–	51.07 (11.67)	125.04 (28.73)
CC-S × MedRisk	B (SE)	–	–	−49.28 (16.59)
	R^2^-change	–	0.15*	0.08
	Total R^2^	0.10	0.25*	0.33*
	Adjusted R^2^	0.04	0.18	0.23

R ^2^ change values in model 2, 5, 8 and 11 are in reference to model 1, 4, 7 and 10, correspondingly. R ^2^ change values in model 3, 6, 9 and 12 reflect the increase in variance accounted for by the interaction term in relation to the preceding model with the main effect for that tract. MedRisk is a composite variable from 0 to 2 where 0 = none, 1 = 1 or 2, and 2 = 3 or more of neonatal medical complications.

* *p* < 0.05.

** *p* < 0.01. PLIC-L, left posterior limb of the internal capsule. PLIC-R, right posterior limb of the internal capsule. CC-G, genu of the corpus callosum. CC-S, splenium of the corpus callosum. B (SE), unstandardized coefficient beta (standard error).

As shown in Table 3, MK values of the left and right PLIC, the genu and splenium of the corpus callosum contributed to language outcomes (Model 2, 5, 8 and 11), explaining an additional 21%, 13%, 16% and 15% of the variance above that accounted for by PMA and MedRisk alone respectively (Model 1, 4, 7 and 10, all *p *< 0.05 or 0.01).

**Table 3:** Prediction of bayley-III language scores at 2 years of corrected-age from tract MK values of PLIC and corpus callosum beyond covariates of post menstrual age at DKI scan (PMA scan) and neonatal medical complications (medRisk).

		Model 1	Model 2	Model 3
PMA Scan	B (SE)	2.94 (1.01)	4.02 (1.87)	3.59 (2.03)
MedRisk	B (SE)	−6.51 (3.62)	−5.83 (2.09)	−11.17 (6.07)
PLIC-L	B (SE)	–	46.86 (11.12)	30.14 (12.75)
PLIC-L × MedRisk	B (SE)	–	–	12.87 (7.13)
	R^2^-change	–	0.21**	0.01
	Total R^2^	0.14	0.35**	0.36*
	Adjusted R^2^	0.08	0.28	0.27
		Model 4	Model 5	Model 6
PMA Scan	B (SE)	2.94 (1.01)	3.81 (1.96)	4.29 (2.06)
MedRisk	B (SE)	−6.51 (3.62)	−3.11 (1.62)	10.26 (4.21)
PLIC-R	B (SE)	–	41.65 (12.17)	82.96 (15.73)
PLIC-R × MedRisk	B (SE)	–	–	−30.29 (10.44)
	R^2^-change	–	0.13*	0.02
	Total R^2^	0.14	0.27*	0.29*
	Adjusted R^2^	0.08	0.19	0.19
		Model 7	Model 8	Model 9
PMA Scan	B (SE)	2.94 (1.01)	4.35 (1.97)	4.27 (2.16)
MedRisk	B (SE)	−6.51 (3.62)	−6.84 (4.23)	−7.95 (6.88)
CC-G	B (SE)	–	47.89 (15.47)	43.79 (14.9)
CC-G × MedRisk	B (SE)	–	–	2.81 (1.51)
	R^2^-change	–	0.16*	0.01
	Total R^2^	0.14	0.30*	0.31*
	Adjusted R^2^	0.08	0.23	0.21
		Model 10	Model 11	Model 12
PMA Scan	B (SE)	2.94 (1.01)	3.39 (1.93)	3.35 (1.91)
MedRisk	B (SE)	−6.51 (3.62)	−1.73 (4.69)	18.02 (7.32)
CC-S	B (SE)	–	56.45 (12.99)	116.31 (25.48)
CC-S × MedRisk	B (SE)	–	–	−39.91 (13.69)
	R^2^-change	–	0.15*	0.03
	Total R^2^	0.14	0.29*	0.32*
	Adjusted R^2^	0.08	0.22	0.23

R ^2^ change values in model 2, 5, 8 and 11 are in reference to model 1, 4, 7 and 10, correspondingly. R ^2^ change values in model 3, 6, 9 and 12 reflect the increase in variance accounted for by the interaction term in relation to the preceding model with the main effect for that tract. MedRisk is a composite variable from 0 to 2 where 0 = none, 1 = 1 or 2, and 2 = 3 or more of neonatal medical complications.

*p < 0.05.

** *p* < 0.01. PLIC-L, left posterior limb of the internal capsule. PLIC-R, right posterior limb of the internal capsule. CC-G, genu of the corpus callosum. CC-S, splenium of the corpus callosum. B (SE), unstandardized coefficient beta (standard error).

**Figure 1:**
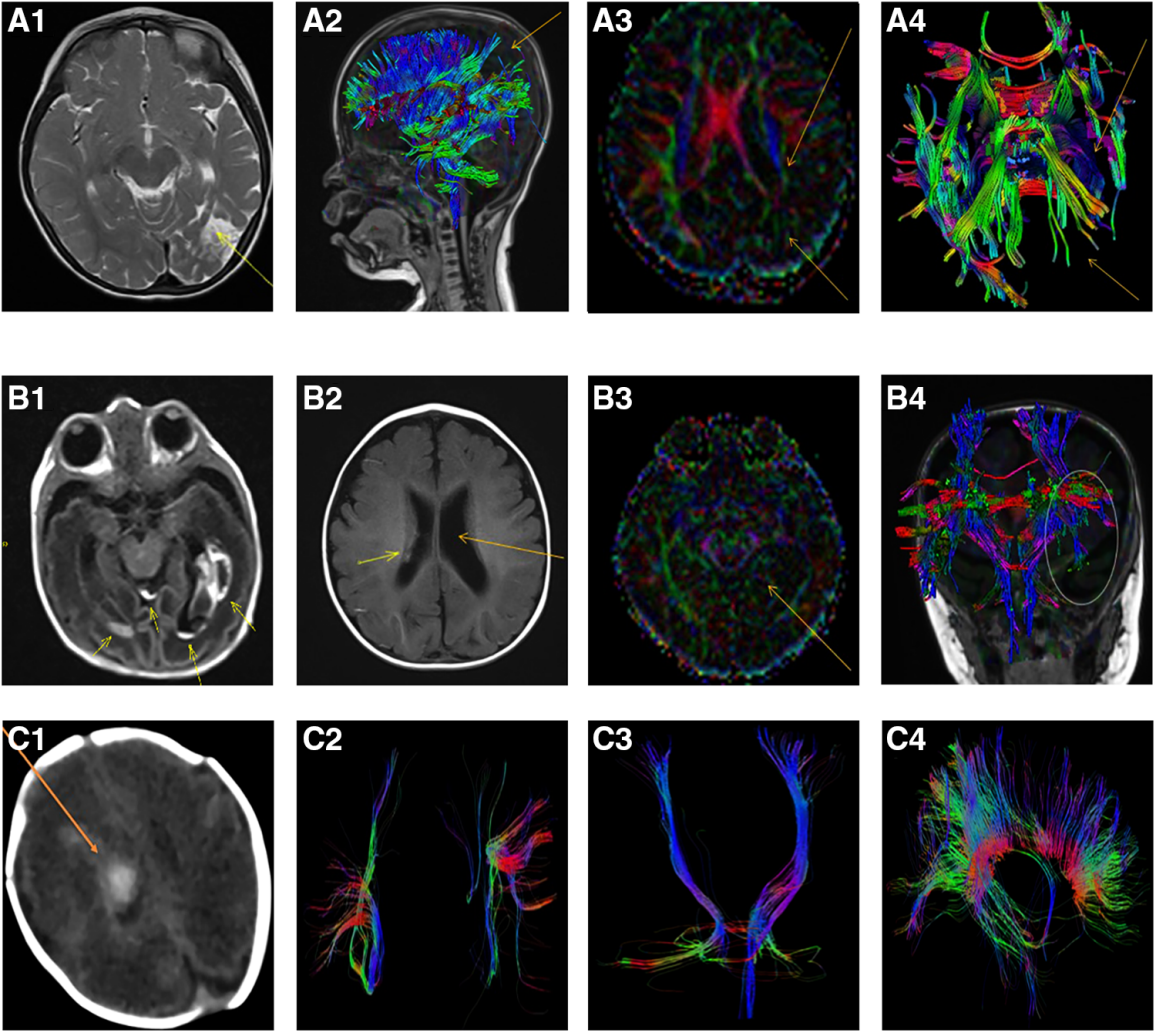
Representative lesions and the losses of nerve fiber bundles from MRI scan and DKI tractography in extremely preterm infants with severe intraventricular hemorrhage. **(A1-A4)**: The loss of nerve fiber bundles in DKI tractography at 36 months after intraventricular hemorrhage of an extremely preterm infants with a gestation age of 27 weeks. **(A1)**: Axial T2-weighted image showed hyperintense in the left temporal and occipital lobes which indicated the formation of softening lesions (arrow); **(A2)**: Sagittal view, DKI tractography showed the loss of nerve fiber bundles in parietal and occipital lobes (arrow); **(A3)**: The decrease and loss of signals of fractional anisotropy (FA) in left hemisphere with DKI scan, especially in the left temporal and occipital lobes (arrow); **(A4)**: Axial DKI tractography showed the loss of nerve fiber bundles in the left temporal and occipital lobes (arrow); **(B1-B4)**: An extremely preterm infants with a gestation age of 27 weeks. **(B1)**: Axial T1-weighted image showed intraventricular hemorrhage and germinal matrix hemorrhage at birth (arrow); **(B2)**: Axial T1-weighted image showed reduced hemorrhagic lesion in the right lateral ventricle and post-hemorrhagic ventricular dilatation of the left lateral ventricle at 6 months old (arrow); **(B3)**: The differential signals of FA between the left and right hemisphere with DKI scan at 6 months old (arrow); **(B4)**: Coronal DKI tractography showed the breakage and loss of nerve fiber bundles in posterior horn of the left lateral ventricle (ellipse); **(C1-C4)**: An extremely preterm infants with a gestation age of 28 weeks. **(C1)**: CT scan showed intraventricular hemorrhage at 12 days after birth (arrow); **(C2)**: DKI tractography showed the right superior longitudinal fasciculus (SLF) became thin and sparse compared with the left SLF at 1 year of corrected age; **(C3)**: DKI tractography showed the right corticospinal tract (CST) became thin and sparse compared with the left CST at 1 year of corrected age; **(C4)**: Corpus callosum was showed by DKI tractography at 1 year of corrected age (Sagittal view).

During the follow-up period, obvious losses of nerve fiber bundles were furtherly observed with DKI tractography (Figure 1).

These results indicated that motor and language scores at 2 years of corrected age positively associated with MK values of the bilateral PLIC, the genu and splenium of the corpus callosum from DKI scan obtained at term equivalent age, after accounting for PMA and number of medical complications.

To provide more evidences, a multivariate logistic regression analysis was next performed with the significant factors on univariate analysis (Supplementary Table S2) and the result was showed in Table 4. We found a 2.5 and 3.3-times increased risk of neurodevelopmental impairment at age 2 in the IVH cases with decreased MK values in the left and right PLIC (OR: 2.507, 95% CI: 1.032–5.927; OR: 3.394, 95% CI: 1.226–6.181, both *p* < 0.05). Similarly, the decreases of MK values in the genu and splenium of the corpus callosum also resulted in significant ORs of 2.143 and 2.099 for development of neurodevelopmental impairment (*p* = 0.041, 0.038). These results confirmed that the decreases of MK values were important risk factors in development of neurodevelopmental impairments.

**Table 4:** Multivariate analysis for risk factors of neurodevelopmental impairments in EPI with severe IVH.

Risk factors	OR	95% CI	*p* value
Decreased MK in the left PLIC	2.507	1.032–5.927	0.029
Decreased MK in the right PLIC	3.394	1.226–6.181	0.021
Decreased MK in genu of CC	2.143	1.324–4.965	0.041
Decreased MK in splenium of CC	2.099	1.107–4.498	0.038
Moderate-severe BPD	2.164	1.084–4.887	0.043
Apgar score at 5 min ≤ 3	2.304	1.005–4.556	0.024

Decreased MKs in the PLIC and CC mean the MK values less than the corresponding cut-off values from ROC analysis. EPI, extremely preterm infants; IVH, intraventricular hemorrhage; PLIC, posterior limb of the internal capsule; CC, corpus callosum; BPD, bronchopulmonary dysplasia; MK, mean kurtosis; OR, odds ratio; CI, confidence interval.

Therefore, the evaluation of white matter microstructural changes with MK values might provide feasible indicators of neurodevelopmental outcomes of extremely preterm infants with severe intraventricular hemorrhage.

## Discussion

Prematurity bears the risk of so many severe complications. In present retrospective study, we aimed to identify microstructural changes of brain white matter by DKI to investigate the related neurodevelopmental outcomes in extremely preterm infants with severe IVH. We found incidences of grade III and IV IVH was 19.4% and 13.3% in extremely preterm infants. The results from diffusional kurtosis imaging demonstrated that the extremely preterm infants who developed neurodevelopmental impairments in later years had lower FA and MK values of bilateral PLIC, genu and splenium of corpus callosum at term equivalent age. Especially, some cases in later childhood were still observed obvious loss of nerve fiber bundles by DKI tractography. These white matter microstructural changes might be pathological foundation of sequential neurodevelopmental outcomes. It has been well documented that the white matter myelination process evolves in a spatiotemporal manner through the brain, roughly from central to peripheral, and dorsal to ventral (20). Mature myelination occurs earlier in the PLIC and corona radiata, followed by the corpus callosum, and finally the lobar white matter. The gestation age of extremely preterm infant is less than 28 weeks and the immature brain is susceptible to severe intraventricular hemorrhage. Damage in the deep white matter involving the corticospinal tract and superior longitudinal fasciculus can lead to motor impairment and cerebral palsy. Moreover, beyond motor impairments, white matter damage also impairs later cognitive ability. Previous researches showed that aberrant FA measures of DTI in the corpus callosum and posterior limb of the internal capsule were associated with poorer language, motor and cognitive abilities in premature children with brain injury from early childhood through school-age (9). As an advanced MRI technique, DKI might provide more accurate parameterization compared with DTI by measuring the MK value within a voxel across different cellular compartments (21). DKI was practicable and effective for investigating ischemic stroke, Alzheimer's disease and epilepsy. DKI might be used to map diffusion properties related to tissue microstructure from language-related white matter tracts. Negative correlations were observed between scores for phonetic memory and MK, axial kurtosis, and radial kurtosis of the left superior longitudinal fasciculus III, which are tracts connecting cortical areas important for phonological working memory (22). An increasing motor activity level was also positively correlated with MK in the inferior, medial and superior longitudinal fasciculus, the corpus callosum and the posterior cingulum in schizophrenia. This association was not found in DTI measures (23). Recently, a few studies validated the advantages of DKI in evaluating brain development in mature and premature newborn brain (10–12).

In this study, we used a serial of linear regressions models to evaluate relations between the white matter microstructure metrics from infant DKI scan and later neurodevelopmental outcomes, controlling for PMA at scan and number of neonatal medical complications. The results indicated that motor and language scores at 2 years of corrected age positively were associated with MK values in the bilateral PLIC, and in the genu and splenium of the corpus callosum from infant DKI scan obtained at term equivalent age, after accounting for PMA and number of medical complications. This could be explained partially by the following analysis. Intraventricular hemorrhage might impair paraventricular white matter lateral to the ventricular bodies including the corticospinal tract and disturb PLIC development. Decreased MK values in the PLIC demonstrates the aberrant myelinization and maldevelopment of the white matters in these regions.

On the other hand, it has been reported that neonatal FA values of DTI scan along the corpus callosum was related to language ability at one year of age (24). Similarly, we think that the decreased MK values reflected the demyelination and maldevelopment of the corpus callosum, which contribute to delays of motor and language. These results also indicated the feasibility and practical value of DKI in evaluating and predicting the neurodevelopmental impairments of extremely preterm infants with severe intraventricular hemorrhage.

In the present study, DKI tractography showed that the superior longitudinal fasciculus and the corticospinal tract became thin and sparse in some extremely preterm infants with severe IVH even at 1 year of corrected age (Figure 1). The results are in line with previous studies. Early anterograde degeneration occurs along the axon direction in the distal corticospinal tract in acute stroke, and can be detected using DKI. Moreover, acute axonal degeneration along the corticospinal tract were correlated with motor deficits (25). A reduction of anisotropy and microstructural complexity in the affected lateral WM bundle of the cervical spinal cord was observed in patients with previous ischemic stroke involving the corticospinal tract. The correlations between DKI metrics and motor performance were statistically significant (26).

With a multivariate logistic regression analysis, we found a 2.5 and 3.3-times increased risk of neurodevelopmental impairment at age 2 in the severe IVH cases with decreased MK values in the left and right PLIC. In the genu and splenium of the corpus callosum, the decreases of MK value also resulted in significant ORs of 2.143 and 2.099 for development of neurodevelopmental impairment. These findings confirmed furtherly that the decreases of MK values were important risk factors in development of neurodevelopmental impairments.

This study is subject to several limitations. Firstly, it went without saying that the limited DKI data was available for this study. Because almost all of extremely preterm infants with severe IVH were in critical condition early in the illness, it was difficult to perform MRI scan immediately. So, a DKI control from the early period of intraventricular hemorrhage was deficient. On the other hand, although we used Readout segmentation of long variable echo-trains technology to increased image quality, it was still limited and could not completely address image distortion. This problem should be furtherly better resolved, especially in the cases with severe IVH lesions. Secondly, we restricted analyses to DKI metrics for PLIC and the corpus callosum likely to be associated with motor and language outcomes of EPI with severe IVH. We did not assess the whole brain, including other nerve fasciculus, to determine the specificity of our findings. Further investigations should be performed to evaluate the entire brain to confirm the findings. Thirdly, it's a retrospective design. Missing data of follow-up restricted the study to complete longitudinal assessment. Fourthly, the sample size for investigating should be furtherly expanded. Adequate number of extremely preterm infants with severe intraventricular hemorrhage were absent for identifying microstructural changes of white matter and evaluating neurodevelopmental outcomes. Moreover, the number of cases in grade II of IVH was high in this study. For avoiding the bias, a multi-center study will be required. Fifthly, although we observed some obvious changes of the superior longitudinal fasciculus and the corticospinal tract of some extremely preterm infants with severe intraventricular hemorrhage by DKI tractography, enough quantitative data still need to be furtherly gathered in the future clinical research.

In conclusion, the rate of neurodevelopmental impairments was considerably high in extremely preterm infants with severe IVH. Abnormalities of PLIC and the corpus callosum were detectable microstructurally using DKI technology. The evaluation of white matter microstructural changes with MK values might provide feasible indicators of neurodevelopmental outcomes of extremely preterm infants with severe intraventricular hemorrhage. Further prospective observations in a larger population of extremely preterm infants are required.

## Data Availability

The original contributions presented in the study are included in the article/**Supplementary Material**, further inquiries can be directed to the corresponding author/s.
