# Supplementary material for: Microstructural changes of white matter assessed with diffusional kurtosis imaging in extremely preterm infants with severe intraventricular hemorrhage
Source: Front Pediatr. 2022 Dec 20;10:1054443. doi: 10.3389/fped.2022.1054443 (PMC9808076; doi:10.3389/fped.2022.1054443)
Supplement: Supplementary file 1 [file Datasheet1.docx]

**Supplementary Materials**

**Supplementary Figure 1**


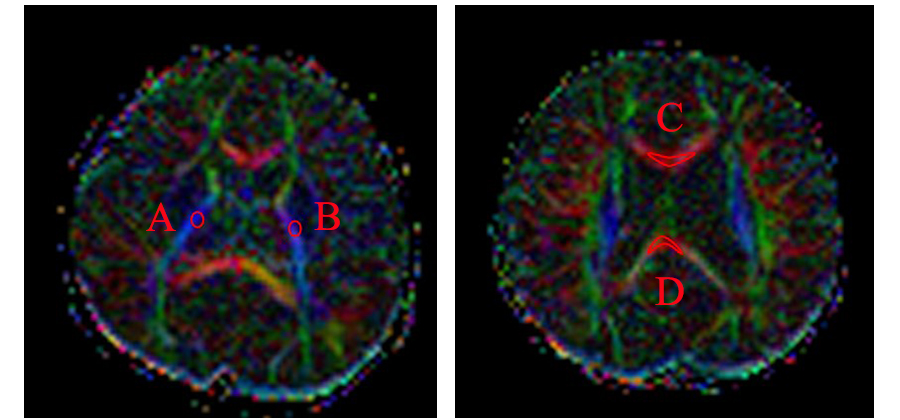


*Position of ROIs on the colored-FA and MK maps. (A): The right posterior limbs of the internal capsule. (B): The left posterior limbs of the internal capsule. (C):* *The genu of the corpus callosum. (D): The splenium of the corpus callosum. ROI = region of interest.*

**Supplementary Table 1.** Intra-observer and inter-observer variability of measurements (n=12).

| ROI |  | Parameters | Intraclass correlation coefﬁcient, 95% CI | |
| --- | --- | --- | --- | --- |
|  |  |  | Intra-observer | Inter-observer |
| PLIC | Left | FA | 0.889, 0.637-0.966 | 0.755, 0.354-0.924 |
|  |  | MK | 0.923, 0.799-0.969 | 0.875, 0.496-0.945 |
|  | Right | FA | 0.915, 0.733-0.966 | 0.809, 0.623-0.959 |
|  |  | MK | 0.935, 0.756-0.978 | 0.926, 0.735-0.981 |
| CC | Genu | FA | 0.931, 0.807-0.988 | 0.854, 0.553-0.961 |
|  |  | MK | 0.974, 0.921-0.994 | 0.952, 0.869-0.983 |
|  | Splenium | FA | 0.853, 0.677-0.984 | 0.744, 0.347-0.919 |
|  |  | MK | 0.954, 0.857-0.989 | 0.945, 0.814-0.973 |

*95% CI, 95% confidence interval. ROI, region of interest.* *PLIC, posterior limbs of the internal capsule. CC, corpus callosum. FA, fractional anisotropy. MK, mean kurtosis.*

Supplementary Figure 2**.**


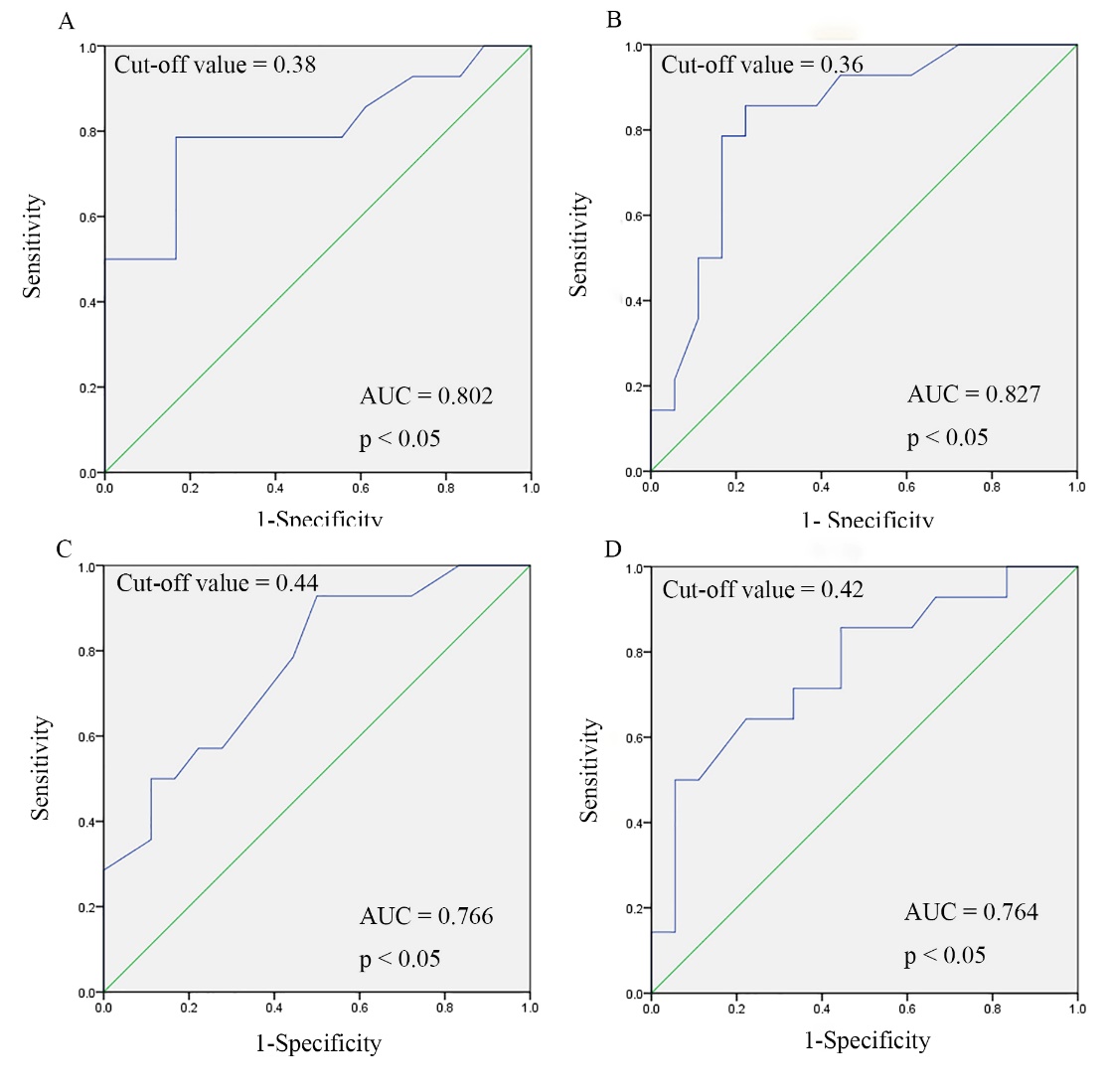


*ROC analysis for the two groups of neurodevelopmental and non-neurodevelopmental impairments. A:* *MK values in the left PLIC; B: MK values in the right PLIC; C:* *MK values in the genu of corpus callosum; D: MK values in the splenium of corpus callosum. PLIC, posterior limbs of the internal capsule. CC, corpus callosum. MK, mean kurtosis. ROC, receiver operating characteristic curves. AUC, area under the ROC curve.*

Supplementary Table 2. Univariate analysis comparing two groups of neurodevelopmental impairments and non-neurodevelopmental impairments in EPI with severe IVH.

| Parameters | Neurodevelopmental impairments  (n=18) | Non-neurodevelopmental impairments  (n=14) | *p* value |
| --- | --- | --- | --- |
| Gestational age (weeks) | 26.19 ± 1.203 | 26.15 ± 1.178 | 0.875 |
| Birth weight (g) | 912.12 ± 151.12 | 932.81 ± 159.19 | 0.558 |
| Apgar score at 1 min | 4.25 ± 1.96 | 4.95 ± 2.12 | 0.040 |
| Apgar score at 5 min | 5.07 ± 2.07 | 6.44 ± 1.62 | 0.023 |
| Invasive respiratory support (n) | 15 | 12 | 1.000 |
| Catecholamine use (n) | 13 | 5 | 0.039 |
| Serum sodium (mmol/L) | 148.01 ± 10.22 | 145.11 ± 5.56 | 0.048 |
| RDS (Grade 3 or 4) (n) | 5 | 5 | 0.712 |
| Hypoglycemia (n) | 9 | 8 | 0.688 |
| NEC (n) | 2 | 2 | 1.000 |
| Moderate-severe BPD (n) | 11 | 3 | 0.025 |
| Retinopathy (n) | 8 | 6 | 0.928 |
| Pulmonary hemorrhage (n) | 2 | 1 | 1.000 |
| PDA (n) | 13 | 9 | 0.712 |
| Early sepsis (n) | 3 | 3 | 1.000 |
| Decreased MK in the left PLIC (n) | 14 | 5 | 0.016 |
| Decreased MK in the right PLIC (n) | 13 | 4 | 0.014 |
| Decreased MK in genu of CC (n) | 11 | 3 | 0.025 |
| Decreased MK in splenium of CC (n) | 12 | 4 | 0.033 |

*Decreased MKs in the PLIC and CC mean the MK values less than the corresponding cut-off values from ROC analysis. EPI, extremely preterm infants; IVH, intraventricular hemorrhage; RDS, respiratory distress syndrome; NEC, necrotizing enterocolitis; BPD, bronchopulmonary dysplasia; PDA, patent ductus arteriosus; PLIC, posterior limb of the internal capsule; CC, corpus callosum; MK, mean kurtosis; OR, odds ratio; CI, confidence interval.*
